# Variation in leaf wettability traits along a tropical montane elevation gradient

**DOI:** 10.1111/nph.14121

**Published:** 2016-07-27

**Authors:** Gregory R. Goldsmith, Lisa Patrick Bentley, Alexander Shenkin, Norma Salinas, Benjamin Blonder, Roberta E. Martin, Rosa Castro‐Ccossco, Percy Chambi‐Porroa, Sandra Diaz, Brian J. Enquist, Gregory P. Asner, Yadvinder Malhi

**Affiliations:** ^1^ Environmental Change InstituteSchool of Geography and the EnvironmentUniversity of OxfordOxford, OX1 3QYUK; ^2^ Ecosystem Fluxes GroupLaboratory for Atmospheric ChemistryPaul Scherrer Institute5232 VilligenSwitzerland; ^3^ Universidad Nacional San Antonio Abad del CuscoAvenida de la Cultura, Nro. 733CuscoPeru; ^4^ Department of Global EcologyCarnegie Institution for Science260 Panama StreetStanfordCA 94305USA; ^5^ Instituto Multidisciplinario de Biología Vegetal and FCEFyNUniversidad Nacional de Córdoba – CONICETC.C. 4955000 CórdobaArgentina; ^6^ Department of Ecology and Evolutionary BiologyUniversity of ArizonaTucsonAZ 85721USA

**Keywords:** cloud forest, contact angle, drip tips, ecohydrology, foliar water uptake, functional traits, leaf hydrophobicity, leaf water repellency

## Abstract

- Leaf wetting is often considered to have negative effects on plant function, such that wet environments may select for leaves with certain leaf surface, morphological, and architectural traits that reduce leaf wettability. However, there is growing recognition that leaf wetting can have positive effects.
- We measured variation in two traits, leaf drip tips and leaf water repellency, in a series of nine tropical forest communities occurring along a 3300‐m elevation gradient in southern Peru. To extend this climatic gradient, we also assembled published leaf water repellency values from 17 additional sites. We then tested hypotheses for how these traits should vary as a function of climate.
- Contrary to expectations, we found that the proportion of species with drip tips did not increase with increasing precipitation. Instead, drip tips increased with increasing temperature. Moreover, leaf water repellency was very low in our sites and the global analysis indicated high repellency only in sites with low precipitation and temperatures.
- Our findings suggest that drip tips and repellency may not solely reflect the negative effects of wetting on plant function. Understanding the drivers of leaf wettability traits can provide insight into the effects of leaf wetting on plant, community, and ecosystem function.

## Abstract 2

See also the Commentary on this article by McDowell & Xu, **214**: 903–904.

## Introduction

Leaf wettability, the amount of water retained on leaf surfaces as a result of dew, fog, and precipitation, is associated with both positive and negative effects on leaf, plant, and ecosystem function. At the scale of the leaf and plant, leaf wetting is often considered to be negative. It inhibits leaf gas exchange and reduces carbon assimilation (Smith & McClean, 1989; Ishibashi & Terashima, 1995; Hanba *et al*., 2004), promotes pathogen growth on leaf surfaces (Huber & Gillespie, 1992; Bradley *et al*., 2003), leaches leaf nutrients (Sase *et al*., 2008), and increases biomechanical stress (Holbrook & Putz, 1989). Alternatively, leaf wetting can have positive effects on leaf and plant function by providing additional water through either the direct uptake of water through leaf surfaces (i.e. foliar water uptake) or the suppression of transpiration. These processes improve plant water status and can contribute to plant growth and survival (Eller *et al*., 2013; Goldsmith, 2013; Gotsch *et al*., 2014). At the scale of the ecosystem, leaf wetting can alter ecohydrology through effects on canopy surface storage, throughfall, and interception loss (Holder, 2012b).

The negative effects of leaf wetting have led to the identification of a number of traits thought to be associated with reducing the amount of water on the surface of the leaf. Specifically, it is assumed that traits related to the leaf surface (i.e. cuticle), leaf morphology, and leaf architecture should positively co‐vary with increasing wetness. The most common leaf surface traits studied with respect to wettability, leaf water repellency and droplet retention, serve as measures of leaf surface hydrophobicity. Thus, high repellency and low retention indicate a reduction of the leaf area covered by water. Leaf water repellency is measured as the contact angle of a droplet of water on a leaf surface (Rosado & Holder, 2013). Water droplet retention, which is sometimes correlated with leaf water repellency, is measured as the leaf angle at which a droplet of water begins to move (Brewer & Smith, 1997; Brewer & Nunez, 2007; but see Holder, 2012a; Matos & Rosado, 2016). Further, the presence of a long, narrow tip on the end of a leaf (i.e. acuminate or attenuate leaf apices), referred to as a drip tip, is a morphology often associated with promoting water loss from leaves and accelerating the drying process (Malhado *et al*., 2012). These surface and morphological traits are in turn modified by leaf architecture, whereby increases in leaf angle (relative to horizontal) can further reduce leaf wettability (Holder, 2012a).

In many instances, leaf traits decreasing leaf wettability have been hypothesized to be ‘adaptive’ (Dean & Smith, 1978; Smith & McClean, 1989; Bradley *et al*., 2003; Burd, 2007; Holder, 2007a; Malhado *et al*., 2012; Meng *et al*., 2014), insomuch as they provide a functional advantage selected for by characteristics of the environment. For instance, frequent leaf wetting associated with high precipitation, compounded by high temperature and relative humidity, may increase leaf pathogen establishment and growth (Ivey & DeSilva, 2001; Harvell *et al*., 2002) and thus would select for traits reducing leaf wettability. However, this assumes that leaf wetting always has negative effects on function. Leaf wetting can clearly have positive effects on function, particularly with respect to foliar water uptake. Is the variation in leaf wettability traits with climate consistent with positive or negative effects of leaf wetting on plant function?

Leaf wettability traits may not vary based on environment alone. There is longstanding recognition that plant functional traits may be constrained by shared evolutionary history (Givnish, 1987). Indeed, if leaf wettability traits are adaptive, this implies that heritability is playing a role and raises the possibility that closely related species may share similar leaf wettability traits. Historically, consideration of the influence of phylogenetic relatedness on patterns of leaf wettability has been limited (Malhado *et al*., 2012; Meng *et al*., 2014). Is there evidence for a phylogenetic signal in leaf wettability traits?

Here we studied how leaf surface and morphological traits associated with leaf wettability, specifically leaf water repellency and the presence of drip tips, vary as a function of climate variables. In particular, if leaf wetting has negative effects on plant function and leaf wettability traits reduce the water on leaf surfaces, then we hypothesize that leaf water repellency and the proportion of species with drip tips will increase as a function of increasing precipitation, temperature, and relative humidity. Alternatively, if leaf wetting has positive effects on plant function through effects such as foliar water uptake, then we hypothesize that leaf water repellency and the proportion of species with drip tips may decrease or not vary significantly with increasing precipitation and that this may be reflected in an inverse relationship between leaf water repellency and the capacity for plants to conduct foliar water uptake. Moreover, if there is a phylogenetic signal associated with leaf wettability traits, then we expect that closely related species will have more similar trait values than expected by chance. To test this, we measured these two leaf wettability traits in > 500 individuals from 150 species occurring in nine tropical forest communities along a 3300‐m elevation gradient in the southern Andes of Peru. In one of the tropical montane cloud forest communities along the gradient, we measured both leaf water repellency and the capacity for foliar water uptake among the most common tree species. The sites we studied vary by > 3000 mm in mean annual precipitation and by > 14°C in mean annual temperature, allowing us to determine how leaf wettability traits change across broad environmental gradients. Finally, we place our findings in the context of a global analysis of previous studies on leaf water repellency.

## Materials and Methods

### Study site

We surveyed a series of 10 1‐ha long‐term forest dynamics plots situated along an elevation transect ranging from 223 to 3537 m above sea level (asl) in the Kosñipata Valley in the southern Andes of Peru (Malhi *et al*., 2010). The plots are part of a comprehensive and ongoing research project coordinated by the Andes Biodiversity Ecosystems Research Group (ABERG; http://www.andesconservation.org) and the Global Ecosystems Monitoring Network (GEM; http://gem.tropicalforests.ox.ac.uk/) and included in the Amazon Forest Inventory Network (RAINFOR; https://www.forestplots.net). The plots range from tropical lowland rainforest to montane cloud forest and were chosen for having minimal evidence of disturbance and relatively homogenous soil substrates and stand structure within each site (Girardin *et al*., 2010). Mean annual temperatures decline linearly along the gradient, spanning a range from 24.4 to 9.0°C. Annual precipitation is high (> 1500 mm) along the entire gradient and demonstrates a strong mid‐elevation peak at *c*. 1000–2000 m asl (> 5000 mm), associated with a front created by cold Andean katabatic winds colliding with moist air brought by the Amazonian Low‐Level Jet (Killeen *et al*., 2007). There is also frequent cloud immersion from 1500 to 3000 m asl, which increases moisture deposition and suppresses evapotranspiration (Halladay *et al*., 2012). Site characteristics are summarized in Table 1.

**Table 1:** Environmental characteristics of 1‐ha study sites occurring along a 3300‐m tropical montane elevation transect, including mean annual temperature (MAT), mean annual precipitation (MAP) and relative humidity (RH)

	RAINFOR site code	Latitude	Longitude	Elevation (m asl)	MAT (°C)	MAP (mm)	RH (%)
Tambopata VI	TAM‐06	−12.8385	−69.296	215	24.4	1900	84.5
Tambopata V	TAM‐05	−12.8309	−69.2705	223	24.4	1900	84.5
Pantiacolla 2	PAN‐02	−12.6496	−71.2627	595	23.5^a^	2366^a^	75.2^a^
Pantiacolla 3	PAN‐03	−12.6383	−71.2744	848	21.9^a^	2835^a^	75.2^a^
San Pedro 1500 m	SPD‐02	−13.0491	−71.5365	1527	18.8	5302	93.7
San Pedro 1750 m	SPD‐01	−13.0475	−71.5423	1776	17.4	5302	93.7
Trocha Union IV	TRU‐04	−13.1055	−71.5893	2758	13.5	2318	86.2
Esperanza	ESP‐01	−13.1751	−71.5948	2863	13.1	1560	89.1
Wayqecha	WAY‐01	−13.1908	−71.5874	3045	11.8	1560	89.1
Acjanaco 1	ACJ‐01	−13.1469	−71.6323	3537	9.0	1980	93.3

a Temperature, relative humidity and precipitation data currently reflect mean of 49‐wk period. asl, above sea level.

### Meteorological data

We calculated mean monthly and annual values of air temperature, precipitation and relative humidity using quality‐controlled and gap‐filled data from weather stations co‐located with plots. Additional details are available in specific studies on the sites TAM‐05 and TAM‐06 (Malhi *et al*., 2014), SPD‐01 and SPD‐02 (Huaraca Huasco *et al*., 2014), TRU‐04 (Girardin *et al*., 2013), ESP‐01 and WAY‐01 (Girardin *et al*., 2014), and ACJ‐01 (Oliveras *et al*., 2014), while climate data from PAN‐02 and PAN‐03 collected in 2013–2014 were processed using the same techniques described for the other sites (Table 1).

### Leaf sampling

Samples were collected as part of the Challenging Attempt to Measure Biotic Attributes along the Slopes of the Andes (CHAMBASA) project in 2013. Based on census data from each plot collected between 2003 and 2013, including tagging, identifying, and measuring all individual tree species ≥ 10 cm diameter at breast height (DBH), a sampling protocol was adopted wherein the species that maximally contributed to plot basal area (a proxy for plot biomass or crown area) were sampled. We aimed to sample the minimum number of species that contributed to 80% of basal area, although in the diverse lowland forest plots we only sampled species comprising 60–70% of the plot basal area. Taxonomy was determined by taxonomists at the Carnegie Institution for Science and voucher specimens are available at http://spectranomics.stanford.edu/species. Within each species, three to five individual trees were chosen for sampling. If three individuals of a given species were not available in the chosen plot, we sampled additional individuals from the area immediately surrounding the plot. Using single rope climbing techniques, we sampled one fully sunlit canopy branch and, where it existed, a fully shaded branch, each at least 1 cm diameter, from each tree. Branches were then maintained in the shade for transport to the lab for immediate leaf sampling. Across all plots, shade branches were sampled from *c*. 40% of all the individuals. Sampling was carried out between April and November 2013.

### Leaf shape morphology

To characterize the presence of drip tips, following Malhado *et al*. (2012), we surveyed one to three photographs of voucher specimens of each species from the field survey and classified the leaves as having (1) retuse, (2) rounded, (3) acute, (4) small tip, or (5) drip tip leaf shape morphology. Species overlap among sites is extremely low; however, in cases where a species was present at more than one site, only photographs from a single site were considered. Photographs were obtained from the Carnegie Spectranomics Data Explorer (http://spectranomics.stanford.edu/species). Leaf shape morphology data are available from the KNB repository (http://dx.doi.org/10.5063/F1J1013H).

### Leaf water repellency

**Figure 1:**
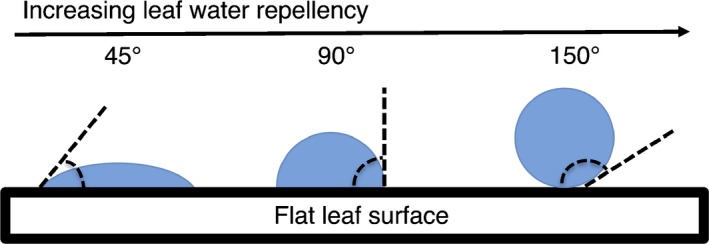
Determination of leaf water repellency by the measurement of contact angle of a water droplet on a leaf surface. Adapted from Aryal & Neuner (2010).

To characterize leaf water repellency, we measured the contact angle of a droplet of water on the adaxial surface of a leaf. We sampled five leaves from each branch collected in the field. Each leaf was first secured flat to a horizontal surface. A 5‐μl droplet of water was then placed on the adaxial side of the leaf using a micropipette and a photograph was taken of the horizontal profile of the droplet using a digital camera. Care was taken to avoid major veins and epiphylls were carefully removed where necessary by hand or using a tissue. We estimate epiphylls affected *c*. 10% of the leaves (Asner *et al*., 2014) and did not differ considerably among sites. While the effects of epiphylls on repellency cannot be excluded (Rosado & Holder, 2013), standardized measurements without epiphylls were considered preferable. The contact angle (also referred to as θ) was measured as the angle between the horizontal line of contact of the water droplet on the leaf surface and the line tangent at the edge of the water droplet (Fig. 1). A larger contact angle indicates higher leaf water repellency (see review of methods in Rosado & Holder, 2013). Before determining contact angle, the water droplet was outlined as an ellipse to aid in more accurate identification of the tangent. Analysis was conducted in imagej v.1.47 (US National Institutes of Health, Bethesda, MD, USA). Leaf water repellency was measured on 502 individuals from 144 different species in 52 families and 93 genera among nine sites along the elevation gradient. Samples were not collected from WAY‐01. Leaf water repellency data are available from the KNB repository (http://dx.doi.org/10.5063/F1J1013H).

### Analysis of leaf shape morphology and leaf water repellency

All statistical analyses were conducted in R 3.2.3 except where otherwise specified (R Core Team, 2015). To determine the difference in leaf water repellency between sun and shade leaves among individual trees we used a linear mixed‐effects model with canopy location as a fixed effect and species nested within site as a random effect. All further analysis was carried out with the average leaf water repellency of the sun and shade leaves measured for a given individual.

To determine the relationship between leaf wettability traits and environmental variation, we considered the traits both as unweighted means at each site and as weighted by summed basal area at each site (for a description of weighting, see Supporting Information Methods S1). These community‐weighted means can provide additional insights as to whether the basal area (a proxy for leaf area; Calvo‐Alvarado *et al*., 2008) of a particular species with a particular trait value can in turn affect other community or ecosystem properties (Violle *et al*., 2007).

The relationship between the proportion of species with drip tips and climate was evaluated using a generalized linear model with binomial errors. The relationships between unweighted and basal area‐weighted leaf water repellency and climate were evaluated using multiple linear regressions. As climate variables may be correlated (i.e. collinearity), we first assessed the pairwise correlation of predictors hypothesized to be important for leaf water repellency (Dormann *et al*., 2013). Based on these results (Fig. S1), we determined that mean annual measures of precipitation, temperature and relative humidity were highly correlated with their respective monthly minimums and maximums. This was not true of maximum relative humidity; however, this measurement is often erroneous at water vapor pressures near saturation (G. R. Goldsmith, pers. obs.). As such, we analyzed only mean annual measures; variance inflation factors were found to be low, indicating that the results are robust (< 1.3). As a consequence of the effects of low sample sizes on statistical power (*n *=* *9–10 sites), we did not include interaction effects. As a result of the statistical analysis of leaf water repellency, we observed that one site (TRU‐04) was particularly influential in the analyses. Therefore, we repeated the analyses without this site, which is subject to considerable cloud immersion (G. R. Goldsmith, pers. obs.). To test whether this cloud immersion could thus be affecting leaf water repellency while also accounting for differences in species composition, we then compared the leaf water repellency of plant species shared between TRU‐04 and a similar site subject to less cloud immersion (ESP‐01) using *t*‐tests.

### Phylogenetics

To determine the presence of a phylogenetic signal in leaf water repellency and leaf shape morphology, we constructed a phylogenetic tree for all species where trait measurements were available. The phylogenetic tree was constructed using the *phylomatic* function in Phylocom 4.2 (Webb *et al*., 2008) using the ‘R20100701’ megatree. Approximate crown ages for each clade were calculated using Phylocom's *bladj* function, with constraints for internal nodes provided by Bell *et al*. (2010), and subsequently corrected for file transcription errors (Gastauer & Meira‐Neto, 2013). We then assigned trait values to the tips of the tree where leaf water repellency was treated as a continuous trait applied using a species level mean and leaf shapes were treated as unordered discrete traits. To determine whether or not values of closely related species were more likely to be similar than expected by chance, we calculated Pagel's λ (Pagel, 1999; Münkemüller *et al*., 2012). Values of λ approaching 0 indicate that the traits are less similar to one another than expected by chance and values approaching 1 indicate that the traits are more similar than expected by chance. To test statistical significance, we applied a likelihood ratio test to compare the likelihood of the estimated λ with the likelihood of a model with no phylogenetic signal where λ = 0. Pagel's λ was calculated using the fitcontinuous (leaf water repellency) and the fitdiscrete (leaf shapes) functions in the Geiger 2.0.6 package available for R (Pennell *et al*., 2014).

To determine the relative contribution of phylogenetic (family, genus and species) compared with individual (within canopy) and environmental (site) effects on the observed variation in leaf water repellency, we also performed a nested variance analysis following Fyllas *et al*. (2009). As a consequence of the sampling design, a similar nested analysis was not possible for drip tips.

### Capacity for foliar water uptake

To characterize the capacity for foliar water uptake as a function of leaf water repellency, we measured three leaves each from three individuals of 12 common species at the site ESP‐01 following the methods in Goldsmith *et al*. (2013). In brief, branches were collected in late afternoon, recut under water and rehydrated overnight. The following morning, a single leaf was excised from the branch and measured for leaf water potential (Ψ_L_) using a pressure chamber. Pressure in the chamber was then slowly increased to −1.0 MPa and maintained for 1 min to induce water deficit. Leaves were then submerged in water for 1 h. Petioles were sealed with parafilm and left above water to prevent water entry. Following submersion, leaves were dried and immediately measured for Ψ_L_. Capacity for foliar uptake was measured as improvement in Ψ_L_ following submersion with adjustment for initial Ψ_L_ given that not all leaves rehydrate to the same Ψ_L_ overnight. Leaves were then measured for leaf water repellency. The total duration of the experiment, including measurements of leaf water potential and repellency, was *c*. 1.5 h per leaf, as leaves were measured and submerged sequentially. Preliminary tests found no evidence that overnight rehydration altered leaf water repellency. The relationship between foliar water uptake and leaf water repellency was determined using linear regression.

### Global analysis of leaf water repellency

To characterize the relationships between leaf water repellency and climate at a global scale, we performed an analysis of published leaf water repellency studies. We carried out a literature search using ISI Web of Knowledge and Google Scholar (15 April 2016) using the term *leaf* AND contact angle* OR *water repellency*. We then looked for additional studies in the literature cited in the relevant studies, as well as the literature citing those studies. To standardize the results, we considered only studies with some representation of the common species of a naturally occurring plant community (at least five spp.) and those that employed a methodology that applied water droplets between 5 and 10 μl in size (Matos & Rosado, 2016). A single study reporting results for plants grown in a controlled environment (i.e. glasshouse) was excluded (Bradley *et al*., 2003). In two instances (Smith & McClean, 1989; Holder, 2007a), similar data sets for a single site were available in more than one study and we retained only one of the two data sets. We determined the mean leaf water repellency of adaxial and abaxial leaf surfaces among species for each location, as well as the mean annual temperature and precipitation where reported. Where climate data were not reported, we used values derived from WorldClim (Hijmans *et al*., 2005) at 1 km^2^ resolution using latitude and longitude provided in the study. Comparable data for calculating relative humidity were not available. As with the analysis for the Peru data set, we determined that mean annual measures of precipitation and temperature were highly correlated with their respective minimums and maximums and analyzed only mean annual measures (Fig. S2).

As we only measured adaxial leaf water repellency for the Peru data set, we used the global data set to assess whether adaxial and abaxial leaf water repellency are related. A strong relationship would indicate the applicability of our results to abaxial surfaces, where stomata are often located in tropical plants (Smith & McClean, 1989). To determine the relationship between adaxial and abaxial leaf water repellency, we used a linear mixed‐effects model with abaxial leaf water repellency as a fixed effect and habitat type as a random effect. To determine the relationship between adaxial leaf water repellency and climate variables, we used a linear mixed‐effects model with temperature, precipitation and their interaction as fixed effects and habitat type as a random effect. For both analyses, adaxial leaf water repellency was log‐transformed before analysis to approximate normality and reduce the heterogeneity of the residuals.

## Results

### Leaf shape morphology

**Figure 2:**
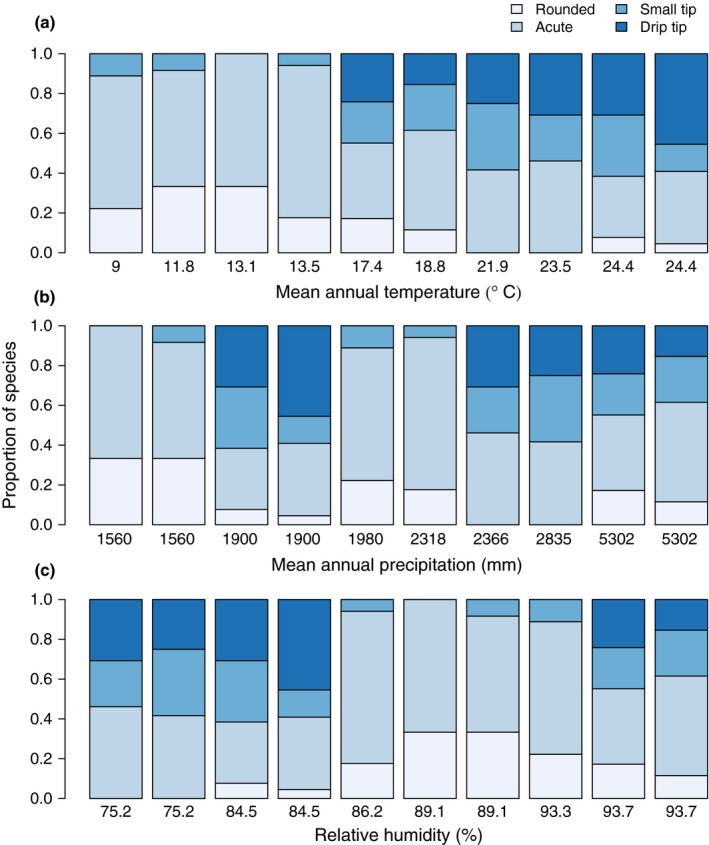
The proportion of species with different leaf shape morphologies as a function of (a) temperature, (b) precipitation, and (c) relative humidity at 10 sites occurring along a tropical montane elevation gradient in the southern Andes of Peru.

Overall, 20% of the 178 species studied had drip tips (Table S1). Of the remaining categories, 19% of the species had small tip leaf shape morphology, 48% had acute leaf shape morphology and 13% had round leaf shape morphology. There were no species with retuse leaf shape morphology. The proportion of species with drip tips increased significantly as a function of increasing mean annual temperature (dispersion = 0.8; *z* = 3.57; *P *<* *0.001), but did not vary as a function of mean annual precipitation or relative humidity (Fig. 2). The increase in the proportion of species with drip tips with increasing temperature corresponded to a decrease in the proportion of species with round leaves.

**Figure 3:**
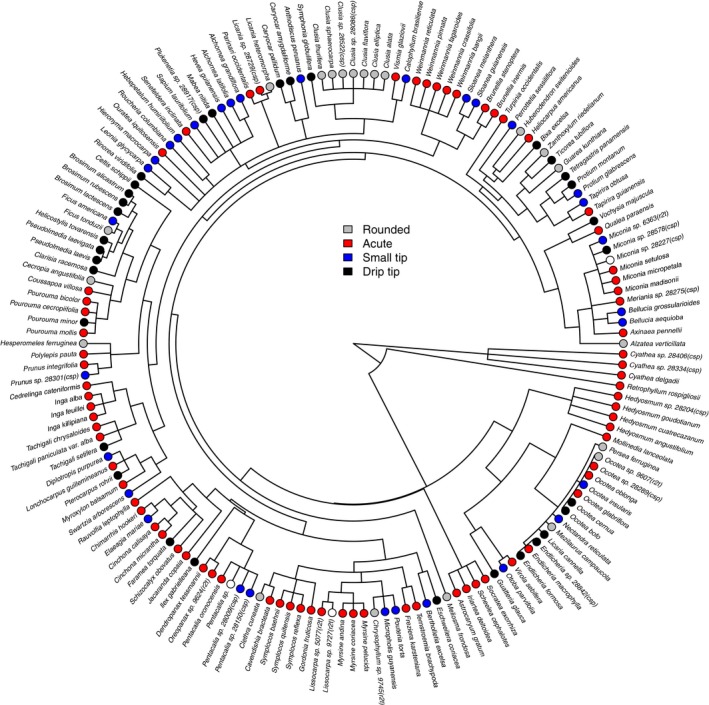
A phylogenetic tree of the species surveyed at 10 sites occurring along a tropical montane elevation gradient in the southern Andes, with each species colored based on a discrete classification of its leaf shape morphology.

As measured by Pagel's λ, there was some evidence for a phylogenetic signal of leaf shape morphologies (λ = 0.72); however, this did not significantly differ from a model without phylogenetic signal (i.e. λ = 0.0; *P *=* *1.0). Species with drip tips were present in 26 different genera (Fig. 3).

### Leaf water repellency

**Figure 4:**
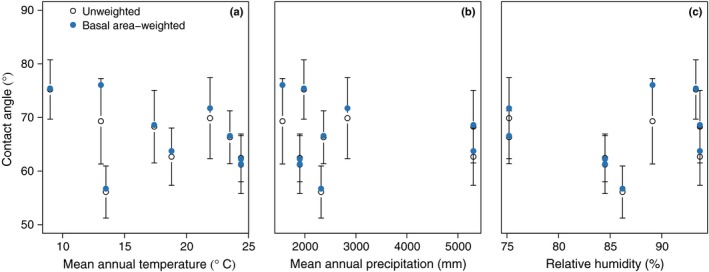
Leaf water repellency (i.e. contact angle) as a function of (a) temperature, (b) precipitation, and (c) relative humidity at nine sites occurring along a tropical montane elevation gradient in the southern Andes of Peru. Data represent mean ± 1 SD.

There was no significant difference in mean leaf water repellency between sun (64.8 ± 7.7°SD) and shade (63.4 ± 7.9°SD) leaves (*F*_1,440_ = 0.1; *P *=* *0.8; Table S2). Site mean unweighted leaf water repellency ranged from 56.1° to 75.2° with an overall mean of 65.7 ± 5.7° (SD), indicating that leaves were highly wettable (*sensu* Aryal & Neuner, 2010). There was no significant relationship between the site‐level mean leaf water repellency and mean annual temperature, annual precipitation, or relative humidity for unweighted or basal area‐weighted means (*F*_1,5_ < 1.3 for each predictor; *P *>* *0.1; Fig. 4). The exclusion of TRU‐04 (the site with high cloud immersion) resulted in a significant negative relationship between unweighted mean site leaf water repellency and temperature (*F*_1,4_ = 23.7; *P *<* *0.01), as well as a significant negative relationship with relative humidity (*F*_1,4_ = 9.6; *P *=* *0.03) and leaf water repellency. These relationships were statistically similar when considering weighted mean site leaf water repellency and excluding TRU‐04. The three species shared between TRU‐04 and ESP‐01, *Weinmannia bangii* Rusby, *Myrsine coriacea* (Sw.) R. Br. ex Roem. & Schult., and *Prunus integrifolia* (C. Presl) Walp., all demonstrated lower mean leaf water repellency at TRU‐04 as compared with ESP‐01, although only the leaf water repellency for *W. bangii* was significantly different between sites (*n *=* *3–5 individuals per site; *P < *0.05; Fig. S3).

**Figure 5:**
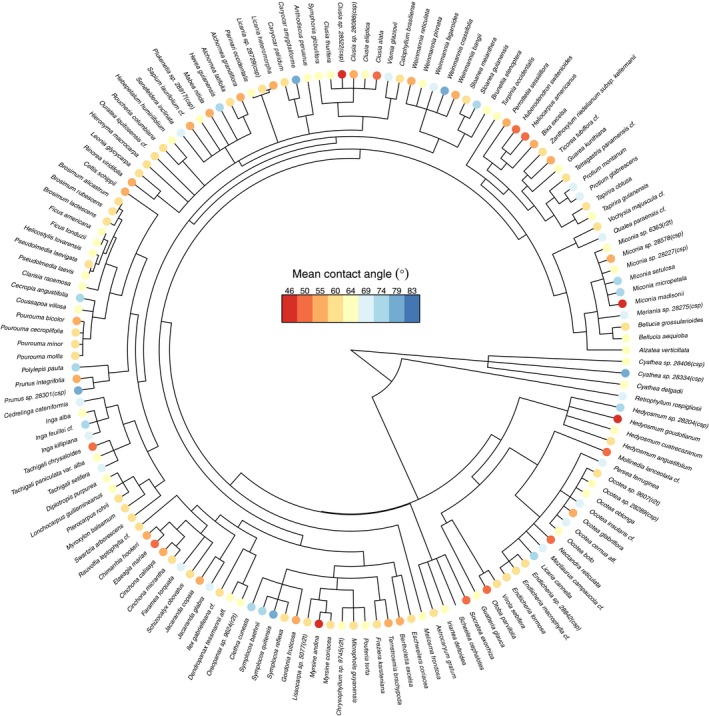
A phylogenetic tree of the species surveyed at nine sites occurring along a tropical montane elevation gradient in the southern Andes, with each species colored according to its mean leaf water repellency (i.e. contact angle).

As measured by Pagel's λ, there was no evidence for a phylogenetic signal of leaf water repellency (λ = 0.0). Species with extreme values of leaf water repellency (range 45.6–83.2°) occurred across the phylogeny (Fig. 5). Some genera were highly variable. For instance, a different species from the genus *Hedyosmum* occurred at each of four sites along the gradient and mean leaf water repellency of the four species varied by nearly 30°.

Variance partitioning also did not provide evidence for a phylogenetic signal. Family, genus, and species accounted cumulatively for only 27% of the observed variance in leaf water repellency, individual (within canopy) effects for 9.0% and environmental (site) effects for 20% (Fig. S4).

### Capacity for foliar water uptake

All the species measured in the tropical montane cloud forest site (ESP‐01) had the capacity to improve their water potential through foliar water uptake (Fig. S5). The capacity for foliar water uptake did not vary significantly as a function of leaf water repellency (*F*_1,10_ = 1.6; *P *=* *0.2).

### Global analysis of leaf water repellency

In addition to the nine sites studied here, we identified eight additional studies with 17 additional sites for inclusion in a global analysis of leaf water repellency (Table 2). Mean annual temperature ranged from 3.8 to 24.4°C and mean annual precipitation ranged from 384 to 5302 mm. Leaf water repellency of the adaxial and abaxial surfaces were significantly related across the global data set, indicating that the results for adaxial leaf surfaces in Peru are also likely to be relevant to abaxial surfaces (*F*_1,355_ = 235; *P *<* *0.001; Fig. S6). Mean adaxial (85.6 ± 38.1° SD) and abaxial (102.0 ± 37.9° SD) leaf water repellency were significantly different (*t* = −10.8; *P *<* *0.001).

**Table 2:** Summary data from a global analysis of published leaf water repellency values; mean contact angle ± 1 SD

Adaxial contact angle (°)	Abaxial contact angle (°)	MAT (°C)	MAP (mm)	Habitat	Latitude	Longitude	Location (no. of species)	Study
86.6 ± 46.5	105.5 ± 48.4	3.9	384	Temperate forest/meadow	41.25	−105.50	USA (34)	Brewer & Smith (1997)
70.5 ± 13.8	83.1 ± 27.5	8.3	442	Temperate forest/grassland	38.89	−104.80	USA (11)	Holder (2012a)
136.6 ± 36.1	153.3 ± 36.7	8.1	750	Steppe	−41.27	−71.33	Argentina (6)	Brewer & Nunez (2007)
78.8 ± 36.7	84.9 ± 32.6	8.1	1550	Temperate forest/steppe	−41.27	−71.33	Argentina (11)	Brewer & Nunez (2007)
48.5 ± 19.5	69.3 ± 32.1	8.1	3000	Temperate rain forest	−41.27	−71.33	Argentina (19)	Brewer & Nunez (2007)
59 ± 9.5	58.3 ± 14.2	na	2200	Tropical lowland forest	−23.52	−45.03	Brazil (5)	Rosado *et al*. (2010)
65 ± 6.1	63.2 ± 7.8	16.1	2000	Tropical montane forest	−23.28	−45.05	Brazil (5)	Rosado *et al*. (2010)
50.6 ± 5.9	84.4 ± 27.7	16.9	1893	Tropical montane cloud forest	15.20	−90.20	Guatemala (12)	Holder (2007a)
74.0 ± 22.8	86.3 ± 34.6	24.4	1002	Tropical dry forest	14.75	−89.50	Guatemala (12)	Holder (2007a)
71.4 ± 5.6	87.0 ± 32.3	10.3	757	Temperate urban forest/meadow	51.00	3.83	Belgium (5)	Kardel *et al*. (2012)
77.5 ± 36.3	105.6 ± 34.4	23.0	1834	Tropical forest	27.57	84.45	Nepal (54)	Aryal & Neuner (2010)
78.6 ± 35.4	97.0 ± 34.2	16.5	1864	Subtropical forest	27.63	85.32	Nepal (60)	Aryal & Neuner (2010)
102.9 ± 31.9	118.8 ± 30.0	8.5	492	Temperate forest	28.77	83.72	Nepal (40)	Aryal & Neuner (2010)
111.1 ± 29.0	121.8 ± 26.5	2.2	418	Subalpine forest/shrub	28.20	85.50	Nepal (42)	Aryal & Neuner (2010)
115.9 ± 31.5	121.8 ± 28.1	2.3	465	Alpine shrub and grassland	28.22	85.57	Nepal (31)	Aryal & Neuner (2010)
49.1 ± 6.0	54.7 ± 13.2	21.0	2500	Tropical lowland forest	−22.95	−43.40	Brazil (7)	Matos & Rosado (2016)
64.9 ± 25.6	72.5 ± 22.4	18.0	2400	Tropical grassland	−22.35	−44.66	Brazil (7)	Matos & Rosado (2016)
75.2 ± 5.5	na	9.0	1980	Tropical montane forest	−13.14	−71.63	Peru (9)	(This work; ACJ‐01)
69.3 ± 8	na	13.1	1560	Tropical montane cloud forest	−13.18	−71.59	Peru (10)	(This work; ESP‐01)
66.3 ± 4.9	na	23.5	2366	Tropical lowland forest	−12.65	−71.26	Peru (13)	(This work; PAN‐02)
69.9 ± 7.5	na	21.9	2385	Tropical lowland forest	−12.64	−71.27	Peru (13)	(This work; PAN‐03)
68.3 ± 6.8	na	17.4	5302	Tropical montane cloud forest	−13.05	−71.54	Peru (29)	(This work; SPD‐01)
62.7 ± 5.3	na	18.8	5302	Tropical montane forest	−13.05	−71.54	Peru (26)	(This work; SPD‐02)
62.5 ± 4.5	na	24.4	1900	Tropical lowland forest	−12.83	−69.27	Peru (27)	(This work; TAM‐05)
61.2 ± 5.4	na	24.4	1900	Tropical lowland forest	−12.84	−69.30	Peru (22)	(This work; TAM‐06)
56.1 ± 4.9	na	13.5	2318	Tropical montane cloud forest	−13.11	−71.59	Peru (17)	(This work; TRU‐04)

na, data are not available.

**Figure 6:**
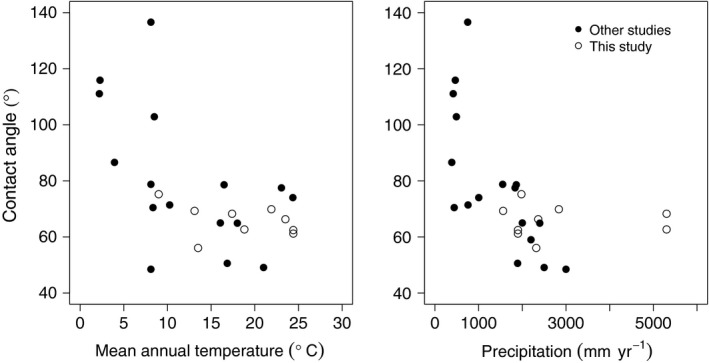
Global data analysis of published leaf water repellency values as a function of mean annual temperature and precipitation.

In the global analysis, leaf water repellency varied significantly as a function of the temperature (*F*_1,6_ = 11.4; *P *=* *0.01), precipitation (*F*_1,6_ = 9.8; *P *=* *0.02), and their interaction (*F*_1,6_ = 7.2; *P *=* *0.04; Fig. 6). In particular, leaf water repellency increased at low mean annual temperature (< *c*. 10°C mean annual temperature) and precipitation (< *c*. 1000 mm mean annual precipitation). The highest mean leaf water repellencies reported are thus in Argentinian steppe and Nepalese alpine shrub/grassland ecosystems.

## Discussion

### Leaf morphology

In contrast to our expectations, we did not observe a significant increase in the presence of drip tips as a function of precipitation or relative humidity. All of the sites along the gradient receive relatively high precipitation and there may be insufficient variation to observe the contribution of precipitation to the presence or absence of drip tips. Recent research on the presence of drip tips across the Amazon suggested that they are not associated with high total annual precipitation, but rather with the precipitation of the wettest trimester, a proxy for the intensity of precipitation (Malhado *et al*., 2012). However, annual precipitation and precipitation of the wettest month are highly correlated in the sites we studied. Neither the amount of precipitation nor the intensity of that precipitation is likely to be the predominant environmental factor driving the observed variation in the presence of drip tips in our study.

Consistent with our expectation that the combination of high temperature and precipitation may increase pathogen establishment and growth, we observed a significant relationship between the presence of drip tips and temperature. Among the sites studied, species with drip tips only occurred where mean annual temperature was > 17°C and the phylogeny demonstrated that sampled genera with drip tips were often exclusive to sites where mean annual temperature was > 24°C. The establishment and growth of bacteria, fungi and epiphylls increase with precipitation and may be compounded by high temperatures (Harvell *et al*., 2002). Drip tips are thought to mitigate such effects by reducing the water on leaf surfaces (Lightbody, 1985; Ivey & DeSilva, 2001). However, the cumulative experimental evidence for drip tips serving this function appears to be equivocal (Ellenberg, 1985; Baker‐Brosh & Peet, 1997; Lücking & Bernecker‐Lücking, 2005; Burd, 2007). It is not clear that the predominance of drip tips in warmer tropical rain forests can be accounted for by pathogen pressure alone.

Ellenberg (1985), conducting a study in Peru, also observed that drip tips were confined to warm tropical rain forests. He hypothesized that they were a function of ontogeny, developing only in high temperatures where no protective leaf bud was necessary and serving as a mechanism for new leaf expansion, then remaining *ex post facto* without a functional role. If this is true, then drip tips may not be an adaptation to reduce the water on leaf surfaces. Instead, they may be a leaf development strategy occurring in some, but not all, warm tropical rain forest genera. However, this does not account for the observation that drip tips appear to be more common on younger compared with older individual plants (Zhu, 1997). This has been hypothesized to be a result of increased exposure of taller canopy individuals to radiation, which increases leaf temperature and promotes drying, thus obviating the need for drip tips and again suggesting their role in promoting the reduction of water on leaf surfaces (Malhado *et al*., 2012).

The presence of drip tips has long been associated with wet evergreen tropical forests (Richards, 1966). Taken together, the current evidence suggests that they are not cosmopolitan, but rather are more predominant in younger and smaller individuals occurring in warmer environments that may be associated with high precipitation intensity. Extending the approaches used here to determine the presence of drip tips among different plant functional types across their ontogeny, in concert with experimental approaches focused on elucidating the relationships between various climate factors and leaf function in plants with different leaf shape morphologies, may further improve our understanding of drip tips.

### Leaf water repellency

In contrast to our expectations, we observed low leaf water repellency (i.e. leaves were ‘highly wettable’) and very low variability among the tropical rain forest sites we studied. Moreover, we found no consistent relationship between leaf water repellency and environmental factors including precipitation, temperature and relative humidity along the gradient, indicating that the proposed negative effects of leaf wetting on plant function are insufficient to select for changes in leaf water repellency. This was true for both unweighted and basal area community‐weighted means of leaf water repellency, such that accounting for the presence of particularly dominant species did not result in significant relationships with the environmental factors studied. Our results, which include some of the lowest mean leaf water repellency values measured to date, are consistent with studies that have identified low leaf water repellency in tropical rain forests with high precipitation (Table 2; e.g. Holder, 2007a; Aryal & Neuner, 2010). When placed in the context of the global analysis, the results demonstrate that high leaf water repellency is actually limited to drier sites (< *c*. 1000 mm mean annual precipitation).

Leaf water repellency, the interaction of a water droplet with the leaf surface, is a function of leaf wax quantity, composition, and structure (Neinhuis *et al*., 2001). These traits may vary among species and thus affect leaf water repellency. As tested by Pagel's λ and the variance partitioning approach, we found no evidence for a phylogenetic signal in leaf water repellency. The properties of the leaf cuticle that drive leaf water repellency do not appear to be shared among closely related species. Bradley *et al*. (2003) observed significant differences in leaf water repellency among 18 different species from the genera *Medicago* and *Trifolium* (Fabaceae) grown from seed in a controlled glasshouse environment. Such results are congruent with the lack of phylogenetic signal observed here.

In natural habitats, leaf cuticle properties are influenced and subsequently modified by interactions with the environment. For example, the high precipitation amounts tropical rain forest leaves experience over their lifetimes may erode leaf waxes (e.g. exposing different wax types), as well as alter their surface structure (e.g. creating a smoother surface), and thus decrease repellency (Neinhuis & Barthlott, 1997). The extent to which this erosion is counteracted by wax regeneration within the lifespan of a leaf is highly species‐specific (Neinhuis *et al*., 2001). The low leaf water repellency observed above *c*. 1000 mm mean annual precipitation may reflect differences in the waxes produced by plants in these environments, or a plateau in the subsequent modification of those waxes above a certain amount of precipitation. This process would also explain the high leaf water repellency observed in dry environments subject to less leaf wax erosion. It has previously been hypothesized that high leaf water repellency benefits ecosystem function in dry environments by decreasing evaporative loss from canopy interception and thus increasing the amount of water that reaches the ground surface (Holder, 2007b). Regardless of the abiotic and biotic processes driving leaf wax properties, our results run counter to the expectation that high precipitation should lead to negative impacts on plant function and thus select for high leaf water repellency. Indeed, our results demonstrate that repellency is consistently very low and that the leaves in the tropical rain forests we studied are highly wettable.

In contrast to precipitation, the effects of temperature on leaf water repellency are not well studied. There was limited evidence for decreasing repellency with increasing temperature along the gradient; however, strong evidence for a relationship between temperature and repellency emerges from the global analysis. High leaf water repellency appears to be limited to colder sites (< *c*. 10°C mean annual temperature). Additionally, a study of leaf water repellency over a temperature gradient of > 25°C in Nepal indicated an increase of *c*. 30° in contact angle with decreasing temperatures, leading to very high repellency at low temperatures (Aryal & Neuner, 2010). This pattern was conserved within a single species (*Juniperus communis*) that occurred along the entire temperature gradient, an indication that environment, rather than biogeography or phylogenetic relationships, drives leaf water repellency. This is contrary to our expectation that high temperature and precipitation should increase pathogen pressure and thus select for high leaf water repellency. At a global scale, the observed increases in repellency at extremely low temperatures may help prevent the formation of frost or ice on leaf surfaces, or (more plausibly for the elevation gradient studied here) reduce metabolically disadvantageous evaporative cooling and thus improve photosynthesis (Aryal & Neuner, 2010).

There was no relationship between leaf water repellency and relative humidity along the gradient, except when considering the data in the absence of the site TRU‐04. The results were similar when considering the relationship between leaf water repellency and vapor pressure deficit (VPD; Fig. S7), a measure of the driving gradient for plant water loss as a function of relative humidity and temperature, rather than relative humidity alone (Rosado *et al*., 2010). Interestingly, there was also no evidence for a significant difference in leaf water repellency between sunlit and shaded branches, where relative humidity may be expected to differ (Aryal & Neuner, 2010). Relative humidity is generally very high along the entirety of the gradient and it is possible that there is insufficient variation to detect a relationship.

In general, plants subjected to a higher VPD (lower relative humidity) have leaves with higher repellency (Koch *et al*., 2006; Rosado *et al*., 2010). Similarly, the surfaces of leaves from cloud forest plants exposed to fog for 3 months became significantly more wettable compared with leaves in the control treatment (Eller *et al*., 2013). This may provide an explanation for the low leaf water repellency observed at TRU‐04, a site where cloud immersion may lead to low VPD (Halladay *et al*., 2012) and where we found some evidence for lower leaf water repellency of species shared between that site and one at similar elevation subject to less cloud immersion (ESP‐01). These findings are also consistent with results demonstrating that foliar water uptake plays an important role in cloud forests (Eller *et al*., 2016) and that high leaf wettability may thus be associated with the positive effects of foliar water uptake. However, as with the findings of Matos & Rosado (2016), we found no relationship between leaf water repellency and the capacity for foliar water uptake. While the negative effects of leaf wetting on plant function do not appear to select for high leaf water repellency in cloud forests, there is still no direct evidence that the positive effects select for low leaf water repellency.

A number of studies have argued that low leaf water repellency (e.g. high wettability) will have negative effects on plant function, such that wet environments should select for high leaf water repellency (Smith & McClean, 1989; Hanba *et al*., 2004; Sase *et al*., 2008). Taken together, the current evidence suggests that high leaf water repellency only occurs in cold and dry environments, while warm and wet environments appear to have low leaf water repellency. Given this, it is intriguing to consider the extent to which leaf wettability has negative effects on leaf function or whether leaf water repellency also reflects the positive effects of leaf wetting for leaf, plant and ecosystem function through mechanisms such as foliar water uptake (Eller *et al*., 2013; Goldsmith *et al*., 2013), although our limited study of foliar water uptake found no evidence for this relationship. Leaf water repellency may not always vary in response to leaf wetting. For instance, high repellency may reflect changes in leaf waxes to prevent cuticular water loss in dry environments or prevent radiation damage in highly exposed environments. Additional research regarding alternative environmental drivers of leaf water repellency may help further our understanding of this trait and its function.

### Conclusions

By assembling extensive data on drip tips and leaf water repellency within and among a series of tropical rain forest communities, we demonstrate that several long‐standing hypotheses about the relationships between leaf wettability and climate do not appear to hold. Specifically, increases in precipitation are not associated with increases in leaf water repellency and the proportion of species with drip tips. We also found limited evidence for a phylogenetic signal in leaf wettability traits, as tested by Pagel's λ, indicating that closely related species do not share similar traits as may be predicted by adaptive hypotheses of function. Instead, our results suggest that leaf water repellency and the presence of drip tips are shaped by a number of different factors. These results imply that the leaf wettability traits we studied either do not serve to reduce the leaf area covered by water or only do so in concert with other (unmeasured) traits such as leaf angle (Holder, 2012a), or that they do reduce the leaf area covered by water, but that the negative effects of leaf wetting for plant function are insufficient to select against high wettability in locations with high precipitation. Alternatively, they may serve other functions entirely, even such that the positive effects of leaf wetting select for increased leaf wettability.

Resolving the effects of leaf wetting on various aspects of plant form, and the extent to which it relates to function, will ultimately involve additional experimental and observational approaches across different spatial and temporal scales. Nevertheless, given projected changes in precipitation in tropical forest ecosystems such as the Amazon (Duffy *et al*., 2015), an improved understanding of the positive and negative effects of leaf wetting on plant, community and ecosystem function remains of great interest.

## Author contributions

The research was designed by G.R.G., L.P.B., A.S., N.S., B.B., R.E.M., S.D., B.J.E., G.P.A. and Y.M. Field data were collected by G.R.G., L.P.B., A.S., N.S., R.C‐C. and P.C‐P. Data analysis was performed by G.R.G. with contributions from B.B. and A.S., with additional input to the interpretation of the results from L.P.B., B.J.E. and Y.M. The manuscript was written by G.R.G. with contributions from L.P.B., A.S., N.S., R.E.M., B.J.E., G.P.A. and Y.M.

## Supporting information

Please note: Wiley Blackwell are not responsible for the content or functionality of any Supporting Information supplied by the authors. Any queries (other than missing material) should be directed to the *New Phytologist* Central Office.

**Fig. S1** Correlation among climate variables for the study sites in Peru.
**Fig. S2** Correlation among climate variables among study sites used in the global analysis.
**Fig. S3** Differences in leaf water repellency for species occurring at two neighboring sites.
**Fig. S4** Partitioning of sources of variance for leaf water repellency.
**Fig. S5** Relationship between foliar water uptake and leaf water repellency.
**Fig. S6** Relationship between adaxial and abaxial contact angles among plant species.
**Fig. S7** Relationship between leaf water repellency and vapor pressure deficit among sites.
**Table S1** Summary of leaf shape morphologies among the study sites
**Table S2** Summary of mean leaf water repellency among the study sites
**Methods S1** Methods used for community‐weighted analyses.
