# Supplementary material for: Variation in leaf wettability traits along a tropical montane elevation gradient
Source: New Phytol. 2016 Jul 27;214(3):989–1001. doi: 10.1111/nph.14121 (PMC5412938; doi:10.1111/nph.14121)
Supplement: Supplementary file 1 — Fig. S1 Correlation among climate variables for the study sites in Peru. Fig. S2 Correlation among climate variables among study sites used in the global analysis. Fig. S3 Differences in leaf water repellency for species occurring at two neighboring sites. Fig. S4 Partitioning of sources of variance for leaf water repellency. Fig. S5 Relationship between foliar water uptake and leaf water repellency. Fig. S6 Relationship between adaxial and abaxial contact angles among plant species. Fig. S7 Relationship between leaf water repellency and vapor pressure deficit among sites. Table S1 Summary of leaf shape morphologies among the study sites Table S2 Summary of mean leaf water repellency among the study sites Methods S1 Methods used for community‐weighted analyses. [file NPH-214-989-s001.pdf]

New Phytologist

New Phytologist **Supporting Information Figs S1–S7, Tables S1 & S2 and Methods S1**

Article title: Variation in leaf wettability traits along a tropical montane elevation gradient

Authors: Gregory R. Goldsmith, Lisa Patrick Bentley, Alexander Shenkin, Norma Salinas, Benjamin Blonder, Roberta E. Martin, Rosa Castro-Ccossco, Percy Chambi-Porroa, Sandra Diaz, Brian J. Enquist, Gregory P. Asner and Yadvinder Malhi

Article acceptance date: 27 June 2016

The following Supporting Information is available for this article:

Fig. S1 Correlation among climate variables for the study sites in Peru.

Fig. S2 Correlation among climate variables among study sites used in the global analysis.

Fig. S3 Differences in leaf water repellency for species occurring at two neighboring sites.

Fig. S4 Partitioning of sources of variance for leaf water repellency.

Fig. S5 Relationship between foliar water uptake and leaf water repellency.

Fig. S6 Relationship between adaxial and abaxial contact angles among plant species.

Fig. S7 Relationship between leaf water repellency and vapor pressure deficit among sites.

Table S1 Summary of leaf shape morphologies among the study sites

Table S2 Summary of mean leaf water repellency among the study sites

Methods S1 Methods used for community-weighted analyses.

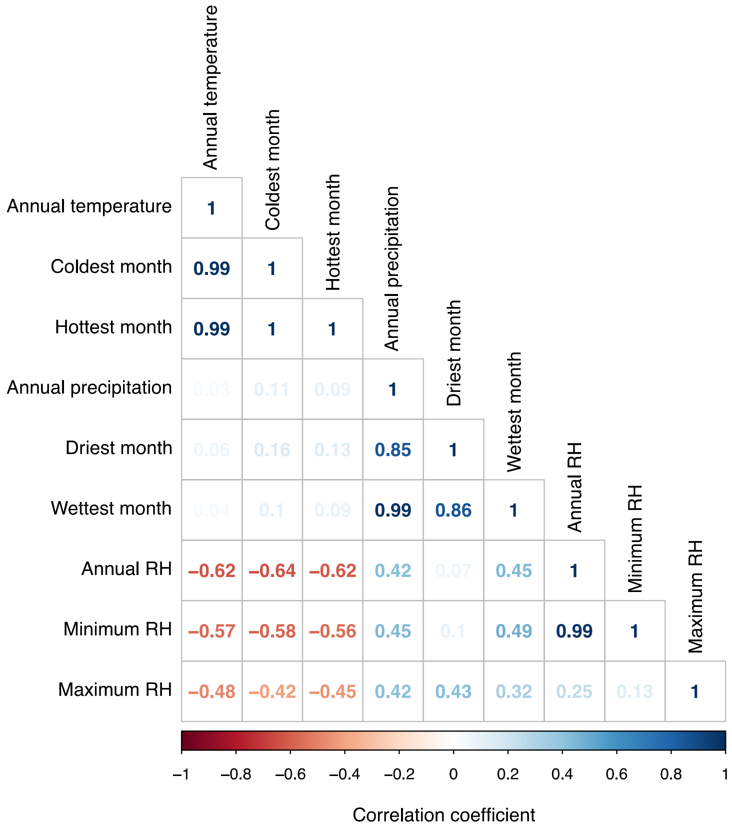

Fig. S1 Pairwise correlation coefficients among temperature, precipitation, and relative humidity at the study sites occurring along a tropical montane elevation gradient in the southern Andes of Peru.

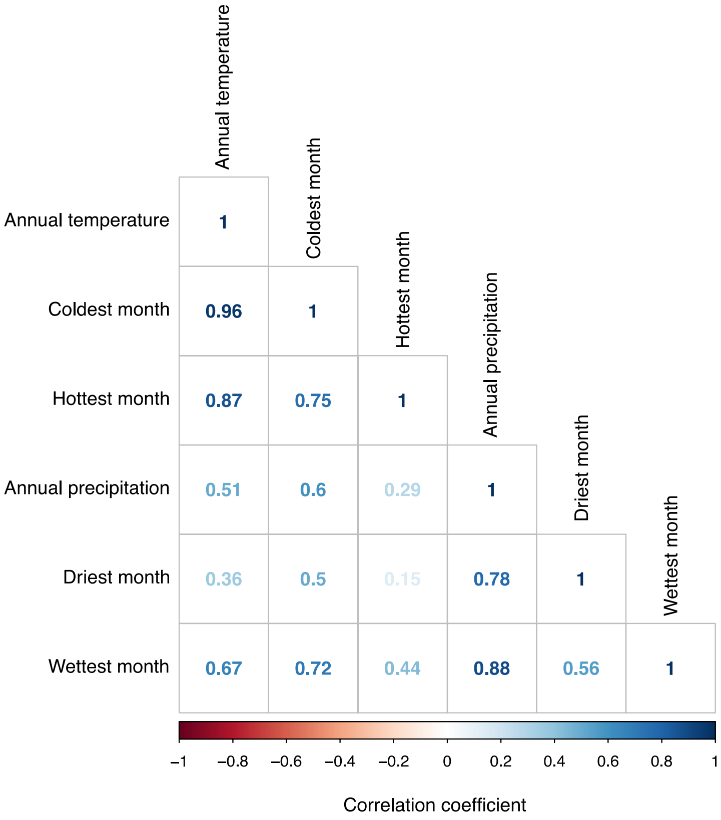

Fig. S2 Pairwise correlation coefficients among temperature and precipitation used in the global analysis of leaf water repellency.

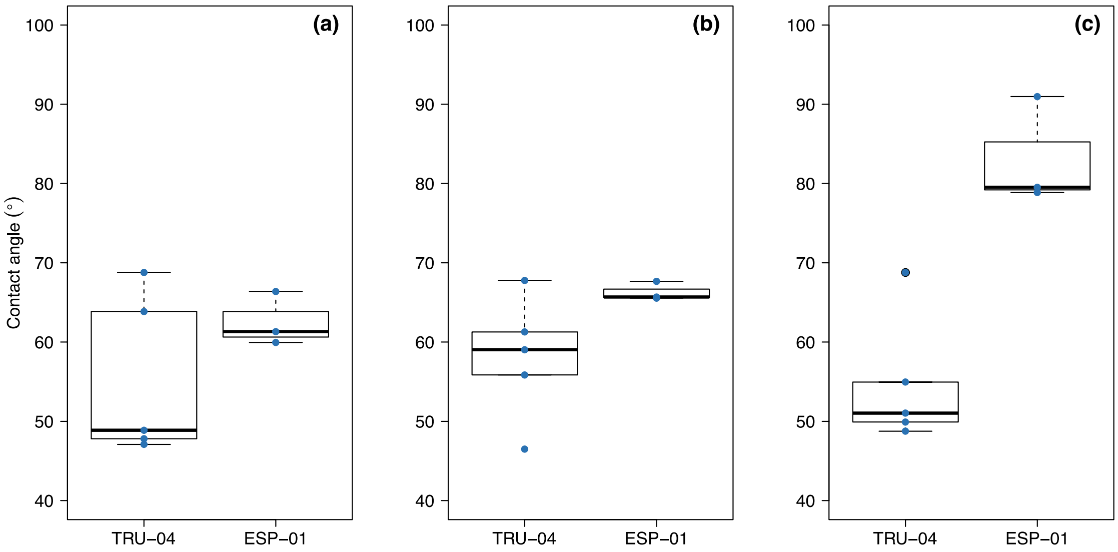

Fig. S3 Boxplots representing differences in leaf water repellency (i.e. contact angle) for individuals of (a) *Prunus integrifolia*, (b) *Myrsine coriacea* and (c) *Weinmannia bangii* occurring at two neighboring tropical montane cloud forest sites occurring along a tropical montane elevation gradient in the southern Andes of Peru. The site TRU-04 is subject to more cloud immersion than the site ESP-01, although they are at similar elevations.

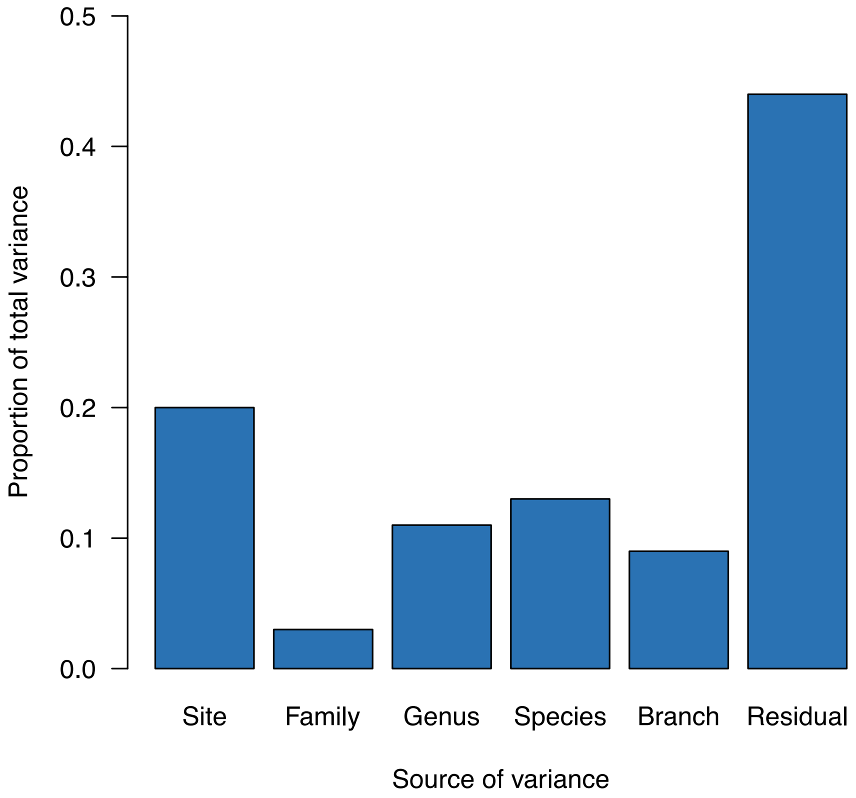

Fig. S4 Partitioning of the total variance observed in leaf water repellency into environmental (site), phylogenetic (family/genus/species), individual (branch), and error (residual) components at nine sites occurring along a tropical montane elevation gradient in the southern Andes of Peru.

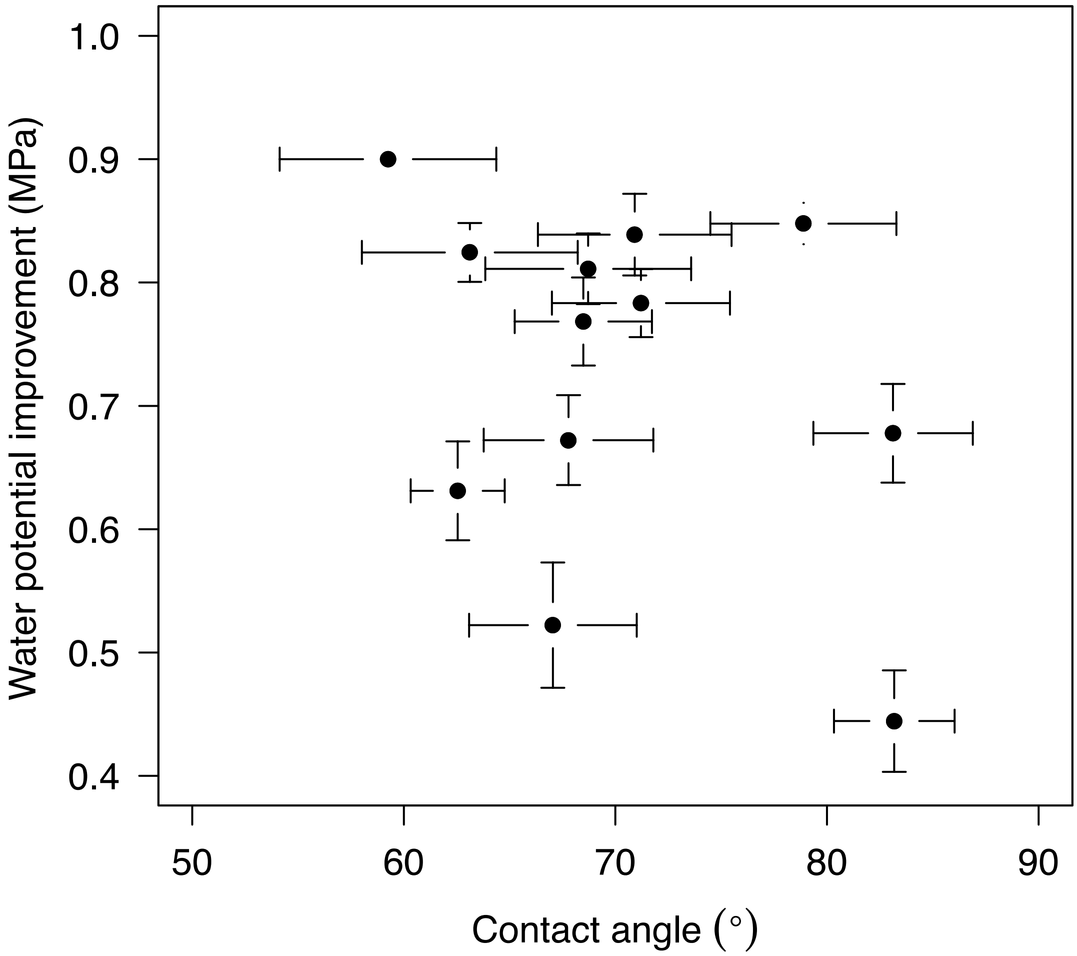

Fig. S5 The relationship between foliar water uptake (measured as water potential improvement) and leaf water repellency (measured as contact angle) among 12 common tree species in a tropical montane cloud forest site occurring along a tropical montane elevation gradient in the southern Andes of Peru. Data represent means ± 1 SE.

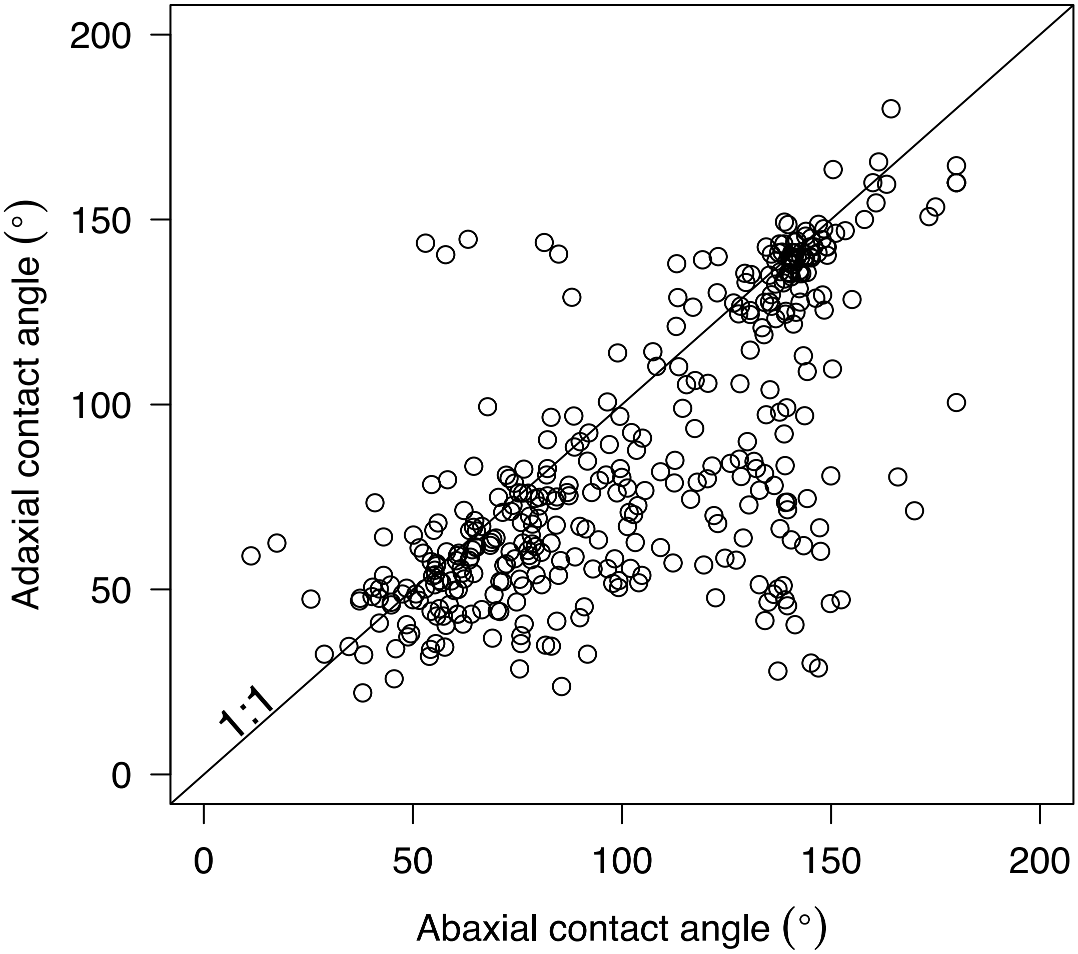

Fig. S6 The relationship between adaxial and abaxial contact angle among plant species from a global data analysis of published leaf water repellency values.

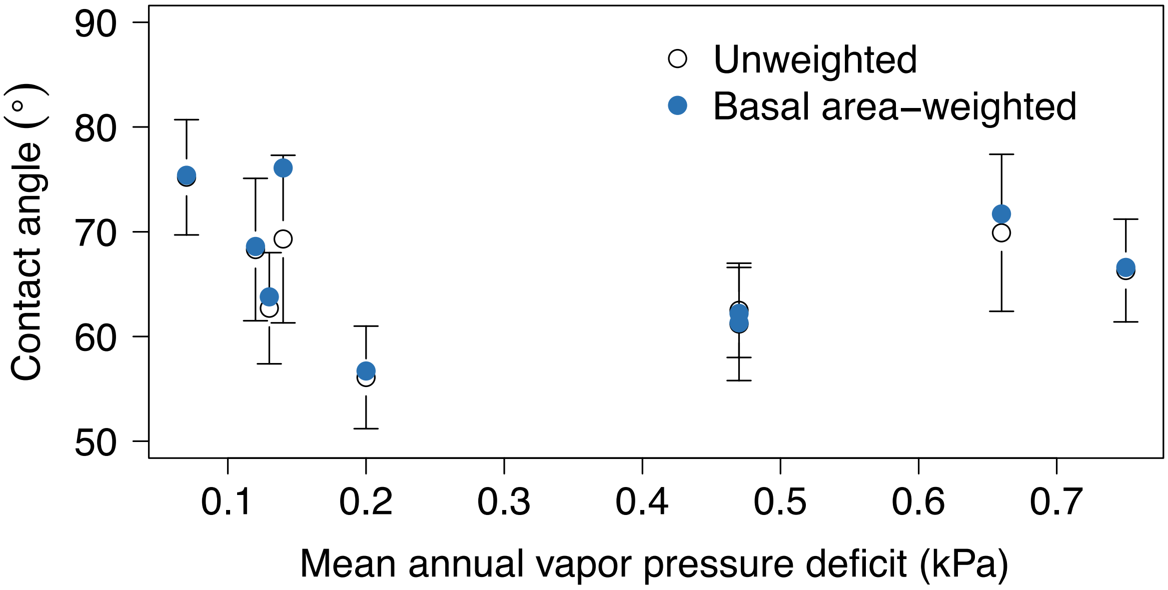

Fig. S7 Leaf water repellency (i.e. contact angle) as a function of vapor pressure deficit at nine sites occurring along a tropical montane elevation gradient in the southern Andes of Peru. The relationship is not significant ($P > 0.1$) unless TRU-04 (where VPD = 0.2) is excluded ($P = 0.01$) – see main text for additional details. Data represent means ± 1 SD.

New Phytologist

Table 1 Counts of leaf shape morphology for species surveyed at sites along a tropical montane elevation gradient in the southern Andes of Peru

RAINFOR site code	Rounded	Acute	Small tip	Drip tip	Total
TAM-06	1	8	3	10	22
TAM-05	2	8	8	8	26
PAN-02	0	6	3	4	13
PAN-03	0	5	4	3	12
SPD-02	3	13	6	4	26
SPD-01	5	11	6	7	29
TRU-04	3	13	1	0	17
ESP-01	4	8	0	0	12
WAY-01	4	7	1	0	12
ACJ-01	2	6	1	0	9

Results are based on surveys of 1-3 photographs of voucher specimens of each species from a field survey and classified as (1) retuse, (2) rounded, (3) acute, (4) small tip, or (5) drip tip.

Table 2 Mean leaf water repellency values for species surveyed at sites along a tropical montane elevation gradient in the southern Andes of Peru

RAINFOR site code	Sun leaves	Shade leaves	All leaves (unweighted)	All leaves (BA-weighted)
TAM-06	60.6 ± 5.2	61.9 ± 6.3	61.2 ± 5.4	61.3 ± 5.2
TAM-05	63.2 ± 4.9	61.1 ± 4.7	62.5 ± 4.5	62.2 ± 4.7
PAN-02	67.7 ± 5.4	62.3 ± 6.1	66.3 ± 4.9	66.6 ± 4.2
PAN-03	70.7 ± 8.4	69.0 ± 8.7	69.9 ± 7.5	71.7 ± 8.4
SPD-02	62.9 ± 5.2	62.0 ± 8.2	62.7 ± 5.3	63.8 ± 5.4
SPD-01	67.8 ± 7.1	67.6 ± 6.5	68.3 ± 6.8	68.6 ± 7.0
TRU-04	55.5 ± 5.6	55.6 ± 4.9	56.1 ± 4.9	56.7 ± 5.3
ESP-01	69.3 ± 8.0	na	69.3 ± 8.0	76.1 ± 11.1
ACJ-01	75.4 ± 5.7	76.1 ± 4.9	75.2 ± 5.5	75.4 ± 5.1

Leaf water repellency was measured as the contact angle of a standardized droplet of water on the adaxial surface of a leaf. Data is presented for mean repellency of sun leaves, shade leaves, sun and shade leaves together (all leaves), and all leaves weighted by the basal area of each species in the plot where it was sampled. Mean contact angle ± 1 SD. na, data is not available.

Methods S1 Methods used for community-weighted analyses.

For community-weighted analyses, we integrated trait data (collected using taxonomic determinations from the Carnegie Institution for Science) with census data (collected using taxonomic determinations from ForestPlots via RAINFOR). Name standardization was performed in cases when these names did not match. First, we set the true taxon name to the Carnegie name when the ForestPlots name did not exist (e.g. individuals outside of the plot). Second, if one but not both of the names was undetermined at the species level, we set the true name to the fully determined name. Third, if every ForestPlots name was renamed by a Carnegie name, we changed the true name for all ForestPlots names to the Carnegie names; additionally, if the new name replaced only undetermined individuals, a true name of the Carnegie name was given to all undetermined individuals. Fourth, if only some ForestPlots names were replaced by Carnegie names, we replaced the true name for just these individuals.

Weighting was defined as w_{ij}, for each site *i* and for each species $j \leq J_i$, where J_i is the total number of species in plot *i*. We also calculated LWT_{im} and LWT_{im}, the species-at-site mean values of a given leaf wettability trait for the subset of species $\{m_i\} \subset \{1,\ldots,J_i\}$ in plot *i* for which trait data were available.

The weighted mean value of a leaf wettability trait in plot *i* was calculated as:

$$\hat{\mu}_i = \frac{\sum_{m \in \{m_i\}} w_{im} LWT_{im}}{\sum_{m \in \{m_i\}} w_{im}} \qquad \text{Eqn 1}$$

and the weighted SD as

$$\hat{\sigma}_i = \sqrt{\frac{\sum_{m \in \{m_i\}} w_{im} \left(LWT_{im} - \mu_i \right)^2}{\sum_{m \in \{m_i\}} w_{im} - \sum_{m \in \{m_i\}} w_{im}^2 \Big/ \sum_{m \in \{m_i\}} w_{im}}} \qquad \text{Eqn 2}$$

Note that because $\left|\{m_i\}\right| < J_i$, these estimators are potentially biased by the leaf water repellency values of species that are in the community, but were unmeasured because their basal area was low. Nevertheless, we expect that the more dominant species measured by our sampling design should most accurately reflect the community's functioning via the mass ratio hypothesis (Grime, 1998).

Reference

Grime JP. 1998. Benefits of plant diversity to ecosystems: immediate, filter and founder effects. *Journal of Ecology* **86:** 902–910.
